# Gaps in measles vaccination coverage in Kasese district, Western Uganda: results of a qualitative evaluation

**DOI:** 10.1186/s12879-022-07579-w

**Published:** 2022-07-04

**Authors:** Abel Wilson Walekhwa, David Musoke, Aisha Nalugya, Claire Biribawa, Godfrey Nsereko, Solomon Tsebeni Wafula, Brenda Nakazibwe, Mary Nantongo, Doreen Awino Odera, Achangwa Chiara, Ross Mathew Boyce, Edgar Mugema Mulogo

**Affiliations:** 1 Department of Community Health, Faculty of Medicine, Mbarara University of Science & Technology, P.O. BOX 1410, Mbarara, Uganda; 2 School of Public Health, College of Health Sciences, Makerere University, P.O. BOX 7072, Kampala, Uganda; 3 School of Biomedical Sciences, College of Health Sciences, Makerere University, P.O. BOX 7072, Kampala, Uganda; 4 Department of Public Health, North Dakota State University, Fargo, ND 58102 USA; 5 Department of Public Health and Hygiene, University of Buea, Cameroon, P.O. Box 63, Buea, Cameroon; 6 Department of Medicine, Division of Infectious Diseases, University of North Carolina at Chapel Hill, Chapel Hill, NC 27599 USA; 7 Ministry of Health, Uganda, P.O BOX 7272, Kampala, Uganda

**Keywords:** Measles, Vaccination, Barriers, Rural district, Uganda

## Abstract

**Background:**

Despite the availability of a highly effective vaccine, measles remains a substantial public health problem in many countries including Uganda. In this study, conducted between June–August 2020 following a local outbreak, we sought to explore the factors that could affect measles vaccination coverage in rural western Uganda.

**Methods:**

We conducted a descriptive study using qualitative data collection approaches in the Kasese district. The research team utilized purposive sampling to identify and select participants from the public health sector and district government. We conducted key informant interviews (KII) and one focus group discussion (FGD). Responses were recorded using portable electronic devices with the FGD and KII guide installed. Interviews were conducted at the health centre and district headquarters. Data was coded and analysed using ATLAS.ti version 8 software through deductive thematic analysis to identify key themes.

**Results:**

Barriers to measles vaccination identified in this study were premised around six themes including: (i) availability of supplies and stock management, (ii) health worker attitudes and workload, (iii) financing of vaccination outreach activities, (iv) effectiveness of duty rosters (i.e., health workers’ working schedules), (v) community beliefs, and (vi) accessibility of healthcare facilities. Respondents reported frequent vaccine supply disruptions, lack of resources to facilitate transportation of health workers to communities for outreach events, and health centre staffing that did not adequately support supplemental vaccination activities. Furthermore, community dependence on traditional medicine as a substitute for vaccines and long distances traveled by caregivers to reach a health facility were mentioned as barriers to vaccination uptake.

**Conclusions:**

Health system barriers limiting vaccination uptake were primarily logistical in nature and reflect inadequate resourcing of immunization efforts. At the same time, local beliefs favouring traditional medicine remain a persistent cultural barrier. These findings suggest an urgent need for more efficient supply management practices and resourcing of immunization outreaches in order to achieve the Uganda Ministry of Health’s targets for childhood immunization and the prevention of disease outbreaks.

**Supplementary Information:**

The online version contains supplementary material available at 10.1186/s12879-022-07579-w.

## Background

Global estimates suggest that approximately 14 million children under 5 years of age live areas highly endemic for measles and 8–12 million children remain unvaccinated [1]. In 2018 alone, models estimate that measles affected nearly 10 million children; resulting in 140,000 deaths globally [2]. The African region remains among the most impacted continents with 1,759,000 total cases and 52,600 deaths reported in 2019 [3], although recent trends continue to show increases in all regions [4]. The increase was particularly pronounced in the WHO Africa Region, however, which recorded a 700% increase in cases over the first 4 months of 2019, highlighting that measles outbreaks remain a substantial and growing public health challenge [5, 6].

In Uganda, measles remains a public health threat. For example, in 2018, 46 of the 146 districts reported outbreaks of the disease [7, 8]. Economically, measles costs over $135,627 per year in societal costs which translates to approximately $44 lost by each affected household per year [9]. The disease has repeatedly ranked among Uganda’s top ten epidemic diseases risks registered by the Ministry of Health (MoH) as reported by the Emergency Outbreak Center (EOC) [10, 11].

The current Uganda National Expanded Program on Immunization (UNEPI) schedule recommends a single measles-rubella vaccination (MRV) administered to infants at 9 months of age. This policy is in contrast to WHO guidelines, which recommend children receive two doses of measles vaccine in the 1st year [12]. Despite the WHO policy Uganda, like many low-income countries, faces logistic and financial barriers to implementation of the two-dose vaccination schedule.

The vaccination program in Uganda is delivered in two ways: (i) a health facility-based model in which caregivers bring their children to the health facility and (ii) outreach programmes in which health workers move to central locations like schools, churches, or market areas to administer vaccines. Community leaders including local council leaders and community health extension workers usually communicate the scheduling of such events to mobilize residents [13]. Participating health facilities may be public or private, but the vaccines are supplied by the government of Uganda which is responsible for the procurement, transport and cost of administration. Uganda has a decentralized healthcare system run by different local governments across the country and there are various levels of these health care facilities ranging from community health worker to health care II, III, IV, general hospital, national referral hospitals, all of which offer vaccination services (Mafigiri, 2021).

From December 2018 through October of 2019, Bugoye Health Centre III (BHC) in Bugoye sub-county, located in the Kasese district of Western Uganda, reported a large number of suspected measles cases [14]. Measles vaccination coverage among children at least 12 months of age for Kasese district and Bugoye HC III in 2018 was 72% and 69% respectively (HMIS, 2018), which is well below the national Ministry of Health targets of 95%. Therefore, our goal was to investigate the barriers to measles vaccination in Bugoye subcounty. To achieve this goal, we purposively interviewed healthcare providers and district leaders who have a direct role in planning and supervising immunization activities. We used a qualitative approach so as to understand the explanations and detailed narratives—in terms of health workers’ and those of district leaders’ perspectives—associated with the observed trends in our prior work, which indicated that female, and older children as well as those not vaccinated were more likely to get measles [14]. Understanding the perspectives of these stakeholders on the barriers to measles immunization among children would inform the planning authorities on strategies to mitigate future measles outbreaks through increased vaccine uptake. Furthermore, this information may be relevant to efforts to increase the uptake of other vaccines.

## Methods

### Study design and setting

**Fig. 1:**
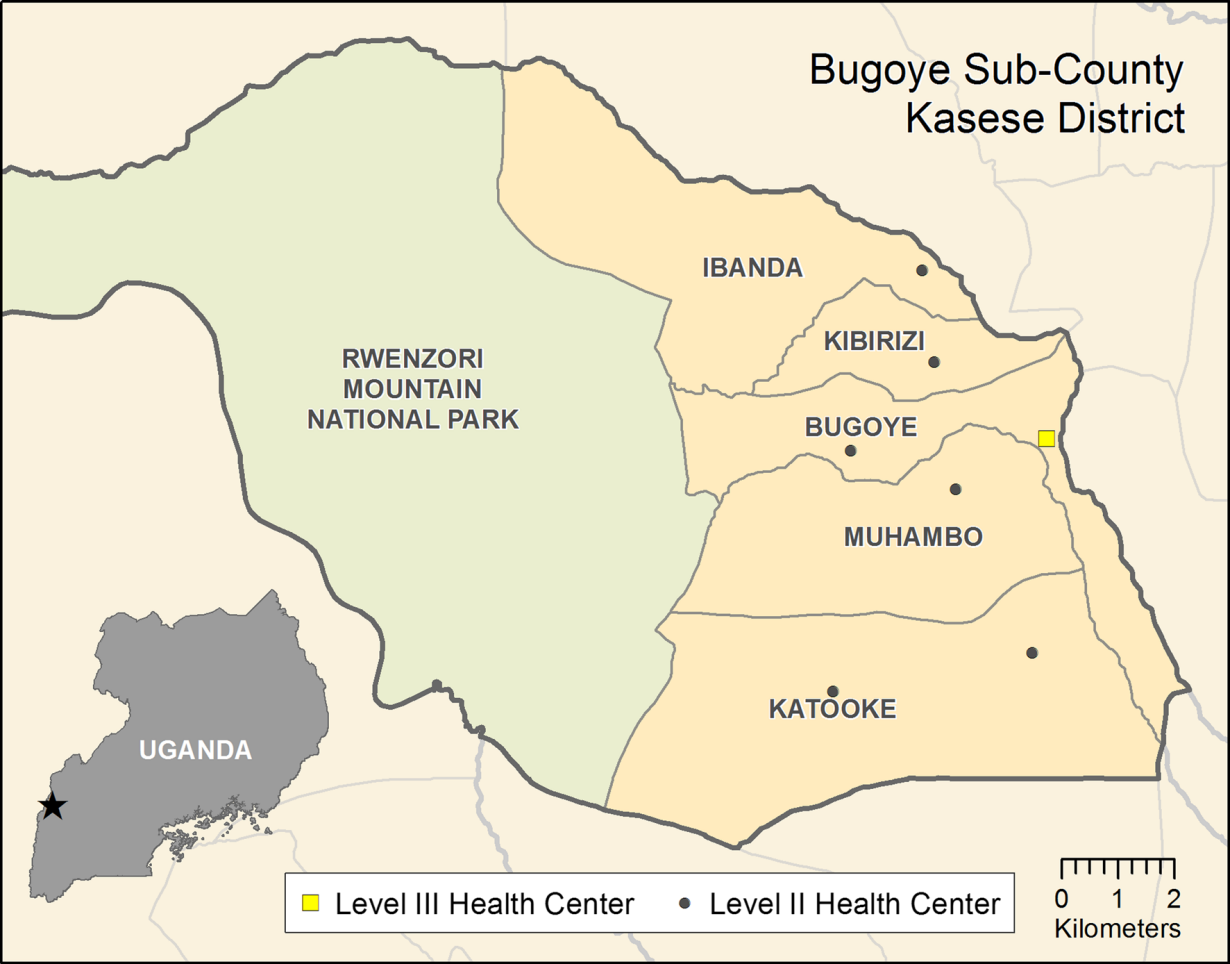
Map of Bugoye subcounty, Kasese district

A descriptive study utilizing qualitative data collection methods was conducted in Kasese district from June 15th to August 7th 2020. Participants included health workers at BHC and officials at Kasese district headquarters. Kasese borders the Democratic Republic of Congo (DRC) as represented in own map drawn (Fig. 1). The sub-county was selected following reports of measles cases presenting to local health centres [14]. Administratively, Bugoye sub-county is comprised of five parishes and 35 villages with a population of approximately 50,000, one-quarter of whom are children under 5 years of age [15]. The sub-county has a total of eight health facilities including seven level II facilities and two-level III health centres, one of which is a private-not-for-profit (PNFP) facility. BHC is the only public level III health facility in the sub-county. Level III health centres are staffed by a senior clinical officer, a clinical officer, laboratory technicians, health assistants, nurses, and midwives. Available services include antenatal care, an immunization clinic, a general outpatient clinic, an antiretroviral clinic for people living with HIV, and small inpatient and maternity wards. The facilities offer immunization services through two models: (i) facility based during week days (e.g., Monday to Friday) and (ii) outreaches (i.e., community-based) which are organized once a month. MRV is available at all vaccination sessions for eligible children (i.e., ≥ 9 months of age).

### Study participants, sampling, data collection and tools

A purposive sampling approach was used to identify, recruit, and enroll study participants. We targeted individuals that play a central role in planning, execution and evaluation of immunization activities at the sub-county and district level. Interviews and focus-group discussions were conducted using KII/FGD guide (Additional file 1), and collected data was analysed through deductive thematic analysis A total of seven key informants, including Resident District Commissioner, District Health Officer, District Expanded Programme on Immunization (EPI) focal person, District Surveillance Officer, In-charge and EPI of BHC and sub-county village health team (VHT) coordinator. Furthermore, a total of seven health workers at BHC were selected purposively to participate in FGD. These included the health workers in maternity and outpatient department (OPD). We elicited responses on the frequency of immunization activities in the area, vaccine supply, interest and competency of the health workers and transport means for immunization supplies. The KII were conducted at the participants’ offices. The FGD were held at BHC after working hours. The participants had been informed about this FGD and they participated in scheduling it on the day they preferred.

### Data management and analysis

Interviews were conducted in English and were recorded on Android-equipped smart phones and tablet device with the KI and FGD guides installed. Each interview recording was transcribed verbatim by the trained research assistant at BHC. For convenience to the participant, the data collection was conducted at their place of work at a time that fitted their schedules. A total of 73 codes for KIs and 17 codes for FGD were initially generated. Additionally, patterns and themes that resulted from the codes that converged and diverged were recorded. The categorized data was used to identify the main themes of the results.

Codes were developed from objective of the study and transcribed data, and then entered into the ATLAS.ti version 8 software for analysis. The software developed codes which were reviewed by the research team and enabled categorization of the study findings. Using deductive thematic analysis, the categorized data was used to develop main themes which made the final results of our study. A total of six key themes emerged from this data analysis. These included; (1) availability of supplies and management, (2) healthcare worker attitude and workload, (3) financial remuneration for vaccination activities, (4) effectiveness of duty rosters, (5) community beliefs and (6) distances to healthcare facilities. These themes were then documented as key outcomes of our study and consequent quotes attached to back up our study findings.

### Quality control and assurance

Research assistants were trained prior to data collection to ensure that they were well conversant with the study protocol, ethics of human subjects research, and data collection methods. The data collection tools were pre-tested with volunteers in a neighboring sub county in order to ensure that they were understood and elicited appropriate responses. The transcripts were then proofread by the corresponding author to ensure fidelity. All nine audio recordings were validated by a member of the research team who had expertise in conducting qualitative studies.

## Results

### Socio-demographic characteristics of study participants

Participants were mostly male (9/14), between 40–49 years of age (6/14), and had attained at least an ordinary diploma (6/14). The majority of these participants had worked for over 20 years in the Uganda civil service (7/14), and identified with the Anglican religion (Table 1).

**Table 1:** Socio-demographic characteristics of study participants

Variable	Attribute	Frequency (n = 14)
Sex	Male	9
	Female	5
Age category (years)	30–39	3
	40–49	6
	50 and above	5
Education level	Ordinary secondary school level	2
	Certificate level	2
	Ordinary diploma	6
	Undergraduate degree	3
	Master’s degree	1
Years in service (years)	0–9	3
	10–19	4
	20 and above	7
Religious preference	Anglican	10
	Seventh Day Adventist	2
	Catholic	2

A total of KI-73 codes and 17-FGD codes were generated. These generated 13 convergent sub-themes and four divergent sub-themes (Additional file 2). Upon thorough review by the research team, a total of 6 key themes emerged (Table 2).

**Table 2:** Summary of themes and subthemes

Codes generated for both key informants and FGDs	Sub themes that emerged	Top six themes
KII-73 codes FGDs-17 codes	13 convergent themes 04 divergent themes	(1) Availability of supplies and management, (2) Healthcare worker attitude and workload, (3) Financial remuneration for vaccination activities, (4) Effectiveness of duty rosters, (5) Community beliefs and (6) Distances to healthcare facilities

The results are presented under six key themes including; availability of supplies and management, health workers’ attitude, lack of financial remuneration for vaccination activities, ineffective duty rosters, community beliefs and long distances to healthcare facilities.

### Availability of measles vaccines and supplies management

Whereas some participants reported that vaccines are always available at the healthcare facilities and district store, a large number mentioned that stock-outs were common. It was also noted that some refrigerators in health facilities were faulty, which adversely affected the storage of the vaccines. In addition, some participants reported that storage space is a major challenge that limited the ability to maintain sufficient supplies of vaccines at health facilities or district stores.

*“I remember vaccines were present but of course on some one or two days, we would have stock outs which was mostly due to limited space for storing the vaccines, such as fridges. In some instances, the fridges are faulty which also contributes to the stock-outs.”* (Female Healthcare worker, BHC)

*“…We have vaccine stock outs and power shortages on some days of the months, especially during the rainy season. As a result, a health worker cannot conduct vaccination outreaches in this place. Solar power is not enough to run the EPI fridge since it’s big. We end up not conducting outreaches.”* (Healthcare worker, BHC (KI 111).

### Health workers’ attitude

The study revealed that the health workers generally had a negative attitude toward engaging in vaccination activities. Respondents noted that due to the low staffing and high volume of work at the healthcare facility, vaccination activities were lower priority.

*“Our services were okay and people still like them. Bugoye Health Centre is on top of our map as a district. However, the attitude of health workers is at 50%. Health workers are demoralized due to the overwhelming work. Sometimes staff are few at the facility and vaccination is done by those few.”* (District Health Team member (KI VI)

*“I will strongly say the poor attitude of health workers and heavy workload hinders vaccination in health centres”* (Healthcare worker, BHC (KI 111)

### Lack of financial remuneration for vaccination activities

It was noted that the lack of transportation hindered community outreach activities. Respondents mentioned that outreaches were often cancelled due to lack of transportation. Furthermore, it was suggested that inadequate funds were allocated to support other elements of community outreaches.

*“The communities were used to us picking the vaccines and finding them in the designated outreach areas. However, some outreaches did not happen during that time because we lacked transport to go and visit those places.”* (District Health Team member (KI 1)

*“What I know is that we had vaccines in stock but the challenge was that the motorcycle responsible for transporting staff was not available, so no outreaches were conducted.”* (District Health team member)

*“Poor health worker financial remuneration…… Immunization activities only rely on Primary Health Care (PHC) funds which mostly cover administrative work. A few outreaches get funds, and even when they do, they are inadequately facilitated”* (Healthcare worker, BHC (FGD 4))

### Ineffective duty rosters at the healthcare facility

Respondents in this study reported that the duty rosters (i.e., schedule of work for the health workers) in place did not specify the team in charge of immunization or allocate health workers to vaccination duty. Duty rosters enable health workers work in a systematic manner ensuring equitable distribution of available human resource to have all services provided in a health facility*.* This could have negatively impacted immunization activities. Effective duty rosters are important for productivity, healthcare worker attendance, employee motivation and performance tracking.

*“…static immunization is done from Monday to Friday here. However, there is no designated team on Immunization and the duty roster doesn’t specify who is to be in EPI for a particular day. This is a challenge.”* (Healthcare worker, BHC)

### Community beliefs

Respondents noted that some communities used locally available herbs as a substitute for vaccines and only resorted to healthcare facilities when the herbs failed to work. This presented a substantial barrier to uptake because these individuals did not interact with health facilities or outreach events.

*“Some community members use herbs as a substitution for vaccines. They still believe in the effectiveness of herbs and only run to the healthcare facility for assistance when these herbs fail to work. This is why they don’t come for community outreaches”* (Male health worker, FGD2 at BHC)

### Long distances to the healthcare facility

The study also revealed that long distances from the healthcare facility was a barrier to vaccination. Respondents mentioned that some mothers reside in parishes far away from the healthcare facility which discouraged them from traveling for vaccination.

*“I will strongly say that the long distances moved by mothers especially those that stay in Katooke, Muhamba parishes and others that are far away, is a challenge.”* (Male, healthcare worker BHC (KI 1))

The study participants also suggested solutions to the observed challenges including: (1) increased budget allocation for the health workers, (2) increased supervision for EPI activities by the district health office, (3) conducting immunization activities in the planned outreaches, (4) organizing refresher trainings for health workers for immunization tracking, planning and documentation, and (5) intensifying community sensitization and mobilization for vaccination programmes in the sub-county.

*“There is need to intensity the mobilization and sensitization of the community members and care givers about how they can identify these diseases and outbreaks such that they can report them early.”*, FGD3

*“There is need to intensify the mobilization and sensitization of the community members and care givers about how they can identify these diseases and outbreaks such that they can report them early”*, FGD3

*“Increase financial remuneration for Immunization activities” KI4*

*“I would strongly recommend for more supervision of immunization activities in the district”.KI3*

*…“yes, the issue of fridges and storage system needs to be looked into” KI3*

*“We need to improve supplies to ensure constantly there, ensure we maintain cold chain more so use of solar system in most of the facilities as it reliable” KI2*

*“continue with intensive mobilization for EPI activities using VHTs including outreaches and even static, maintain the tracking system for the mothers and children who could be missing on the antigens, continue using the LC1s strategy” KI1*

*“Need for capacity building of health workers in data management, also emphasize follow up and tracing of the mothers who are due to Measles vaccines and other antigens since some children disappear, develop a system of following all children from the time of birth to a time of last doses including Vitamin A and deworming. Conduct mass measles campaigns in this sub county to bring services nearer to the people here”. KI4*

## Discussion

Our study highlights the health system barriers that affect measles vaccination coverage including inconsistent vaccine stocks, inadequate cold chain storage, missed opportunities for community outreach, lack of dedicated transportation and relatively low prioritization of immunization activates among staff. Overall, these findings suggest an urgent need to better organize and resource vaccination activities in order to achieve set vaccination targets in rural areas of Uganda.

Vaccine stock outs were reported as one of the primary barriers to effective delivery of immunization services in the study area. Stock outs occur when vaccine supplies are depleted either arising from an unexpectedly high number of recipients or the orders underestimate the eligible underlying population. Many times, the children affected by stock outs are those who are already underserved due to distance to health facility. Vaccine stock outs have an established impact on immunization outputs as the children who are expected to get such vaccines at such slated times often miss out waiting for the delivery and distribution of the vaccines [16]. Some of the challenge of vaccine stock outs could also be due to the small amount of storage capacity that some facilities have, including at the district level, which is responsible for distribution of the vaccine. Another factor contributing to the vaccine stock outs could be the delays by the national supply system through the National Medical Stores (NMS) to supply vaccines. This impediment could also be coupled with the inefficiant management at both the facility and the District Local Government where requisitions could take long to reach NMS culminating into delays. Regardless of the source, caregivers experience frustration after traveling long distances only to be told that the vaccine is not available. Such experiences may also contribute to lower enthusiasm for future care seeking due to lack of trust in the vaccine supply.

In addition, the UNEPI policy of unpreserved vaccine which must be discarded at the end of the session or 6 h after reconstitution, should be modified so as to optimize this vaccine’s availability, such that vials can in practice be opened whenever an eligible child presents. This policy has direct contribution of vaccination practice at health facility level for example, the ordering of vaccine in 5-dose rather than 10-dose vials increases the health workers’ hesitation to open a vial for fear of being blamed for wastage, thereby reducing missed opportunities and raising timely coverage. These health workers tend to request caretakers to either wait for others or to go and come back on a particular where they anticipate more numbers of children to be vaccinated such that the opened vials serve more children. This is a common phenomenon in low- and middle-income countries which have limited budget allocation for vaccines but also low technological access that limits the planning and prediction of such supplies [17]. This study findings are in line with other studies conducted at Mulago National Referral Hospital, Uganda that showed that majority of the new borns miss immunization due to vaccine stock outs [18, 19]. This calls for budget increase for EPI activities at the district health offices, and proper planning by stakeholders like national medical stores (NMS), ministry of health and district health department to minimise the occurrence of stock outs [17].

The above factors outlined issues of vaccine supply. However, barriers also exist among the health care workers themselves, ranging from description of burn out, increased workloads, and inconsistencies in pay. Low prioritization of vaccine services among health care workers was one of the reported barriers in our study. Multiple health care workers reported having lost their motivation to work, while those that were working were the ones that needed the money despite being burned out. We note that this study was conducted during the first wave of the novel coronavirus pandemic, which may have exacerbated these issues. During this period health workers stretched their routine activities to support COVID-19 response. For example, some staff were redeployed to other health facilities that needed additional support. With some staff leaving due to burn out, the staff who have stayed are more reliant on pay to stay engaged. The sign of exhaustion among health workers was realized as many participants kept pointing out their frustrations arising from non-payments due to delays in release of Primary Health Care (PHC) funds. Although increased funding would be helpful, other non-financial incentives and improvements should also be explored. Staff performance could be motivated by promotions, further studies, more capacity building sessions. Our findings are in line with other studies done in Uganda that showed that health workers’ attitude is critical for better immunization activities [20, 21]. The district leadership and management of BHC should explore various non-financial motivation mechanisms for their staff such that there is continuity of service delivery even in absence of PHC funds. Furthermore, there is need to draw out a clear work schedule for the health facility staff spelling out when they will be working in the EPI Section.

The absence of transport during the time when the district reported measles outbreak [14] was reported as a hindrance to increasing the accessibility of immunization activities. Movement of health workers from their respective health facilities to different outreach sites was difficult. Participants reported negotiating credit with local private transport companies including commercial motorcycle drivers, while awaiting the release of PHC funds for use as a refund. According to Ministry of Health EPI guidelines, the transportation of vaccines from the district vaccine store to lower health facilities is the mandate of the district health office which can be achieved if transport means and fuel is availed through PHC funds [22]. Our study revealed that this transportation of vaccines was affected due to lack of available transport facilities. This could be possible due to technical delays in the release of PHC funds which affects the fuelling of available vehicles. Transportation challenges could also be associated with diversion of EPI vehicles—commonly referred to as “GAVI vehicles”—for other administrative duties in the district hence affecting the immunization activities. Uganda being a low-income country, resources such as vehicles are shared by the district leaders depending on the priority demand at a particular time.

The rugged terrain of the area greatly limits the accessibility of some villages. Many caregivers cannot climb to high terrain areas where the outreach sites are located which greatly contributes to children missing their scheduled vaccinations thus increases the numbers of unvaccinated thus increases the chances of measles outbreaks. This finding is in line with other studies conducted in Uganda where it was clearly noted that physical barriers like hilly/mountainous geographical locations and road terrain greatly limited the accessibility of services for children under 5 years [13, 20, 21, 23]. This calls for geographical context planning for immunization services to cater for such difficulties such that outreach sites could be located in such places where critical need is known.

The findings showed that less attention was given by health workers to tracking the missed opportunities for measles immunization even when children were taken to the health facilities for other services. Health workers are few and the idea of tracking the vaccination status of children during routine service provision is not taken as a priority. In the same vein, there are no specific reminders to different caretakers on their scheduled dates although the community health workers normally remind many caretakers in totality on the need to go for health services in various health facilities. In addition, caretakers and caregivers forget to bring their eligible children for the measles vaccination at 9 months. This could be attributed to inconsistencies in the payment of staff for immunization activities, which (as reported by various stakeholders) results in less prioritization of those immunization activities. This poor remuneration of these health workers could also explain why many of them were reported to have lost interest in EPI activities leaving this to the community health workers (locally referred to as Village Health Teams) who may not be technical enough to track the missed children. Furthermore, the health workers also asserted that their large workload reduces their ability to concentrations on tracking children who miss particular vaccines, including the measles vaccine. This study finding is in agreement with previous studies done in similar regions (e.g., Hoima district) that showed that staff overload affected the attention to immunization activities which contributed to measles outbreak [13]. There should also be deliberate efforts to identify the children who missed the vaccination through review of health facility records, follow up of caregivers of children who are due for MRV leveraging local leaders and public communication (i.e., radio) to remind the caregivers about the need to complete the vaccination for their children. Furthermore, reminding caregivers of the importance of maintaining immunization cards may prove useful, as these cards show the dates for subsequent visits.

Our study has a number of strengths. We interviewed the staff who are directly involved in the provision of measles vaccination services which helped to understand real issues they face. We also targeted both the stakeholders in management positions and those of the implementing staff which, which method gave a broad picture to the perceived barriers. The study also employed two methods (e.g., KIIs and FGD) that helped to deeply explore the subject matter. Lastly, we captured ideas and potential solutions to improve measles vaccination coverage that stakeholders can utilize. However, this study also had limitations including the relatively small number of interviews and FGDs, which was primarily due to the fact this was a graduate student research project with a modest budget. We also interviewed only one VHT who was already involved in other activities at BHC and his views many have not be representative of other VHTs in communities. We also did not interview caregivers so our results reflect only those of the service providers, rather than the end-users. Lastly, we did not interview the Ministry of Health and NMS staff who could expand on issues related to vaccination supplies and budget allocation for the district.

## Conclusion

Logistical issues and inadequate resources, including vaccine supply, clinic staffing, and transportation for outreach events, represent substantial barriers to effective delivery of vaccination services in rural western Uganda. In addition to the solutions proposed herein, we recommend further study of (i) the existing surveillance capacities in the district that could be leveraged to identify unvaccinated children, (ii) perceptions of caregivers and caretakers on vaccination programmes in the district, (iii) novel interventions to remind caregivers of vaccination schedules.

## Supplementary Information

**Additional file 1.** Key informant/FGD guide.

**Additional file 2.** Summary of subthemes.

## Data Availability

All data supporting our findings are contained in the paper. There are no restrictions to data sources, however, details of the full data may be accessed through the corresponding author; Mr. Abel Wilson Walekhwa, Email: wabelwilson@gmail.com.

## Abbreviations

BHC: Bugoye Health Centre III
DHO: District Health Officer
DSO: District Surveillance Officer
EPI: Expanded Programme on Immunization
MoH: Ministry of Health
PHC: Primary Health Care
UNEPI: Uganda National Expanded programme on Immunisation
UVRI: Uganda Virus Research Institute
VHT: Village Health Teams
WHO: World Health Organization
