# Supplementary material for: Gaps in measles vaccination coverage in Kasese district, Western Uganda: results of a qualitative evaluation
Source: BMC Infect Dis. 2022 Jul 4;22:589. doi: 10.1186/s12879-022-07579-w (PMC9251590; doi:10.1186/s12879-022-07579-w)
Supplement: Supplementary file 1 — Additional file 1. Key informant/FGD guide. [file 12879_2022_7579_MOESM1_ESM.docx]

# ADDITIONAL FILE 1: KEY INFORMANT/FGD GUIDE

1. Comment about measles coverage in Bugoye Subcounty in 2018.

2. Talk about the service availability (where, when, etc)

3. Comment about the availability of vaccines at time of outbreak both at district and health facility level

4. Talk about mobilization for immunization at Bugoye HCIII and the entire district

5. Talk about the Human resource for immunization (adequacy, motivation) of Bugoye HCIII and Kasese district health department in 2018 and how do you think it links to measles vaccination?

6. Comment about surveillance system of Bugoye HCIII and Kasese District

7. Comment about the Facilitators and barriers to immunization in district and sub-county

in Bugoye HC III.

9. What should be done to improve the trend of measles coverage in Bugoye Sub County.

Thank you so much for your attention and your time.

Do you have some questions for me?.
