# Supplementary material for: Gaps in measles vaccination coverage in Kasese district, Western Uganda: results of a qualitative evaluation
Source: BMC Infect Dis. 2022 Jul 4;22:589. doi: 10.1186/s12879-022-07579-w (PMC9251590; doi:10.1186/s12879-022-07579-w)
Supplement: Supplementary file 2 — Additional file 2. Summary of subthemes. [file 12879_2022_7579_MOESM2_ESM.docx]

**Additional file 2: Summary of Subthemes**

| **Types of theme** | **Subthemes** | **Quotes** |
| --- | --- | --- |
| Convergent | 1.Availability of vaccines | *“The vaccines were at district level but the challenge was transport to go and pick them from the store. We didn’t have a motorcycle”.FGD1*  *“I remember vaccines were present but of course on some one or two days, we would have stock outs which was mostly due to limited space for storing the vaccines, such as fridges. In some instances, the fridges are faulty which also contributes to the stock-outs.”* (Female Healthcare worker, Bugoye HC III)  *“What I know is that we had vaccines in stock, but the challenge was no outreaches conducted” KI2* |
|  | 2. Availability of all immunization logistics (cold chain boxes, ice, cotton wool, swabs, weighing machines) | *“Some supplies are got from the district. But sometimes we request for vaccines from the district and it can out of stock and you find that they are also waiting from National Medical Stores (Ministry of Health) in Kampala to supply to them and sometimes we can find like a week or 3-4 days without conducting Immunization activities”*. FGD2  *“...We have vaccine stock outs and power shortages on some days of the months, especially during the rainy season. As a result, a health worker cannot conduct vaccination outreaches in this place. Solar power is not enough to run the EPI fridge since its big. We end up not conducting outreaches.*” (Healthcare worker, Bugoye Health Centre III (KI 111)  *“yes, the issue of fridges and storage system needs to be looked into”* KI7  “….*Now some of the health facilities near BHC have no fridges yet getting transport to come for vaccines could be challenging”.* KI3 |
|  | 3. High motivation of health workers for Immunization activities | *…”Haa here we have strong VHTs who help to refer mothers to come here. Even the health assistant while in the community, he tells them about immunization services at the facility” FGD4*  *..”Our mobilizers are ourselves the staff and the vaccinators, but our money is not paid”* FGD3  *“Poor facilitation at the health facility and only relies on PHC which mostly covers administrative works, this covers few outreaches which are also inadequately facilitated” FGD2*  *“Increase facilitation for Immunization activities” FGD3*  *“The biggest issue was attitude of the health workers to go for these outreaches”.KI2*  *“I think we should even be at 100% in that place. Because we even have partners who support EPI activities and very health worker in the district wants to go and work there”.*KI4 |
|  | 4. Workload among health workers | *“We have only 04 vaccinators and yet we have other duties under ICCM” FGD5*  *“We are few as some health workers don’t want to be put on duty of immunization. They don’t even pay us our allowances and it becomes very hard for you to die alone with too much work” FGD6*  *“our staffing is very okay in BHC. By the way that place is among the best staffed health facilities in the district”.KI3* |
|  | 5. Availability of trained Community health workers | *“We are very few and not happy. May be because the money for immunization activities is very little. Therefore, let them increase facilitation for Immunization activities,” Because if we have enough facilitation, it’s when we can run out activities well” FGD 7*  *“Our services were okay and people still like it. BHC is on top of our map as a district. Attitude of HWs is at 50%, the health workers would be enjoying their work of immunization but demoralized by work as they are overwhelmed, sometimes staff a are few at the facility and done by them”KI4*  *“Some health facilities have always shifted vaccines to another health facilities that have sound fridges” KI3*  *“Here in the district we use VHTs and also we use local council leaders like LC III, LC1 and they help in bringing the children for immunization*” KI6 |
|  | 6.Competence of health workers to implement Measles vaccination activities | *“Also the health workers were using VHTs to vaccinate and this immunization wasn’t taken seriously by the health workers, also poor mobilization for the vaccination”*KI3  *“but also some mothers easily forget and also health workers in those facilities don’t check on child immunization cards and am not sure if they forget or they just don’t have that time”* KI5  *“What I know is that health workers are enough here and are motivated just that they have not been doing EPI activities on daily basis, they could not do it on daily basis due other competing activities like ANC and postnatal”.* KI5  *“I will say PHC funding was available, RBF (results-based financing) from Enabel programme was present so could motivate the staff” KI5* |
|  | 7. Functionality of fridges at BHC and lower health facilities. | *“Storage capacity is a big challenge (Fridges are few), Some fridges are faulty in some health facilities, so we are congesting some health facilities thus congesting it”* KI4 |
|  | 8. Community beliefs | *“Here people love immunization and normally take their children for vaccination” FGD4*  *“Some people are still refusing vaccines and use local herbs” FGD1*  “*Actually in Bugoye sub county we have no problem with parents perceptions about vaccination but the challenge must have been on for mobilization for the children in the communities, also there the health workers’ attitude towards immunization”* KI3  “*I have never heard of the use of local herbs in Kasese district, maybe you educate me on this”* KI2  *“Some just come to come and get the ANC number from here just in fear of anything that may happen during delivery otherwise many still deliver in communities, something happens during immunization Programmes they can begin well and end on DPT3 and they disappear and fail to turn up for the measles vaccination at 9 months”KI5* |
|  | 9. Tracking of unvaccinated children at OPD | *“There is need to intensity the mobilization and sensitization of the community members and care givers about how they can identify these diseases and outbreaks such that they can report them early”. FGD2*  *“The mobilization for Immunisation was mostly done by VHTs”* KI3  *“I will say this outbreak is due to reluctance of mothers to bring children for immunization”* KI2  *“But also we have a tracking system in the sub county of Bugoye where the health facility keeps track of the mothers through the assistance of local council leaders*” KI3  *“Here our people live a nomadic lifestyle more so during a season for digging. Mothers move and go to stay in distant places temporarily”.KI4*  *“….but the issue is that some caregivers take their children to complete the last antigens like measles vaccine in lower health centre II and we can’t know if they went or not” KI5* |
|  | 10. Effectiveness of traditional medicine against measles | *“Some communities also use Herbs in substitution to vaccines, some community members still believe in herbs and when these herbs fail to work is when they run to facility for assistance” FGD1* |
|  | 11. Management of duty rosters at BHC | *..”the challenge is we are so busy in maternity and you have no time for this”* KI5  *“But static immunization is done from Monday to Friday here. But here there is no designated team on Immunization and the duty roster doesn’t specify who is to be in EPI for a particular day”* KI5 |
|  | 12.Supervision of health workers by district leadership. | *“Inadequate supervision of Immunisation activities due to lack of transport. The GAVI vehicle is available but does most work of the district, supervision of immunization activities is reduced”.KI2* |
|  | 13. distance of health facilities from homes of caregivers | *“Here we have no money for mobilizing the people to come for immunization. For example, there is less community mobilization and sensitization about Immunization services in mostly hard to reach places. By the way some people stay beyond 10 kms” FGD5*  “Long distance to hard to reach areas (Bukyarara, Babata,Magoma)” FGD3  *“We had many children who had missed immunization, also we had outreach sites but not functional and therefore we had missed opportunities and children were not immunized”,*KI3 |
| Divergent | 1.Inadequate Vaccine supplies in the district | *“Some shortages in supplies is the issue, the problem is in the ordering. We order for few vaccines and other supplies”* KI4 |
|  | 2.Unreliable transport means for immunisation activities | *“For outreach activities health workers use Boda boda and foot due to the terrain of the area, the funds are not on available on time and they wait until quarter is over” KI3* |
|  | 3.Continued occurrence of disasters like floods | *“Displacement of people from their paces due to floods, people they stay in camps” KI3* |
|  | 4.Absence of outreaches and some not implemented | *“The communities were used of us picking for them the vaccinations and finding them in designated outreaches but some outreaches were not functional during that time”.* KI1  *“The terrain was a problem, no transport means for EPI activities including transportation of vaccines at Bugoye HC III”* KI3 |
